# The Impact of Narcissistic Leader on Subordinates and Team Followership: Based on “Guanxi” Perspective

**DOI:** 10.3389/fpsyg.2021.684380

**Published:** 2021-07-07

**Authors:** Lin Wang

**Affiliations:** School of Economics & Management, Foshan University, Foshan, China

**Keywords:** narcissistic leader, followership, guanxi, dominance complementarity theory, leadership

## Abstract

Studies have shown that narcissistic individuals are more likely to become leaders in uncertain situations, while few studies paid attention to the relational mechanism, linking a narcissistic leader with subordinates and team attitudes and behaviors. Based on the “guanxi” and dominance complementarity theory, we examined the influencing mechanism of narcissistic leaders on subordinates and team followership (TF). Two-wave data collected from 326 employees in the manufacturing and technology industry in China supported our hypothesized model. We have found that narcissistic leaders have a negative impact on followership (F) and TF of subordinates; Supervisor–subordinate guanxi (SSG) and team leadership relationship identity play a partial mediating role between narcissistic leadership and subordinates and team followership. Furthermore, individual and team values play a moderating role in the process of influencing a mechanism. In other words, the higher the individual tradition and team power distance (PD), the less negative impact of leader narcissism on SSG and team leadership relationship identity. Theoretical and practical implications are discussed. We also offer several promising directions for future research.

## Introduction

Enterprises are facing more uncertainty and following a vital crisis, which put forward new requirements and challenges for corporate leaders. Studies have shown that, compared with a more certain external environment, narcissistic individuals are more likely to become leaders under uncertain circumstances, and they can take more effective actions in crisis management (Nevicka et al., 2013; Watts et al., 2013). Scholars have always maintained a lasting interest in the narcissistic leaders, but, whether the narcissistic leaders are insufferable or attractive still remains inconclusive. Most studies believe that the narcissistic leaders have both “black” and “white” sides, and their impact is complicated and controversial (Huang et al., 2019). Thus, under the background of the current turbulent external environment, it is of practical significance to study the narcissistic leaders, leaders with complex personality traits, and to discuss their influence on subordinates and teams. Scholars such as Owens et al. (2015) also suggest to study the influence of narcissistic leaders from more detailed perspectives. Although previous research discussed the influence of individual-level narcissistic leaders on employees and organizational-level CEOs on organizations (Khan and Mata, 2020), the mechanism of narcissistic leaders in different cultural backgrounds has not yet been fully investigated. And there is little discussion about the influence of the group narcissistic leaders at the team level. Therefore, this study aims to explore the influence of narcissistic leaders on subordinates and team followership (TF) in the context of collectivist culture, further figure out the boundary conditions of their influence, and reveal the complexity of narcissistic leaders.

Bjugstad et al. (2006) believed that it was far from enough to rely solely on the individual will and wisdom of the leader to achieve effective leadership. Effective leadership is composed of the collective wisdom of the team and the effective followership (F) of the leader from numerous followers. Followership is one of the most important inner driving forces for the development of a company, especially in the current complex and turbulent external environment. Previous studies mainly focused on how different team leadership characteristics or behaviors affect the team effectiveness. And they regarded the followers in the team as passive receivers and executors of the command of the leader (Uhl-Bien et al., 2014). The proactiveness of followers in the interaction between the leader and the team is ignored. This also leads to relatively scarce studies, which are based on the perspective of followers to investigate the influence of characteristics or behaviors and TF of leaders (Cao et al., 2019). Thereby, in addition to discussing the influence of characteristics of leaders on employee followership, this study also discusses their influence on team followership. Therefore, this study aims to promote related empirical research on team followership to some extent by exploring the influence of narcissistic leaders on team followership.

Compared with the western contractual relationship based on equal exchange, Chinese leaders pay more attention to “*guanxi*” in their connections with subordinates and teams (Farh et al., 1998). Most of the studies are based on the western contractual relationship of equal exchange, and they investigate the role of leader-member exchange (LMX) in the influence on the attitude and behavior of narcissistic leaders and employees (Liao et al., 2019; Bernerth, 2020), but ignore the influence of “guanxi” between leaders and employees on subordinates and teams. Therefore, this study explores the mediating mechanism of the influence of leader traits on subordinates from the perspective of “guanxi.”

In addition, the dominance complementarity theory believes that dominance complementarity can promote satisfying and effective relationships. When one individual is in a predominant condition and exhibits influence, firmness, and dominance, the other individual is expected to have tolerant, submissive, and negative role behaviors. This complementation can effectively coordinate the behavior and interaction of both parties (Chen et al., 2016). Hence, in order to figure out which subordinates and teams are more compatible with narcissistic leaders, we explored the moderating effect of subordinates and team values on narcissistic leaders.

Especially as a personality variable with a typical Chinese cultural imprint and reflecting individual value difference, individual traditionality (T) has an important influence on the attitude and behavior of employees. Some studies on the management of Chinese enterprises have found that the traditionality of employees has a very important influence on the performance of leadership (Chen and Aryee, 2007), and this characteristic also conforms to the traditional cultures, such as collective conformity and obedience to authority. Traditional culture and power distance (PD) have important moderating effects on the evaluation of organizational support of employees (Farh et al., 2007), while it is found that Chinese cultural values are characterized by high-PD and collectivity (Hofstede and Bond, 1984). The combination of team power distance and different leadership traits will have different influences on team efficacy and team level leadership relationship identity (Hu and Judge, 2017). Therefore, the research aims to explore the role of a specific individual and team values in the process of influence of leaders on employees in Chinese culture so as to expand relevant literature.

In general, this study explored the influencing mechanism and boundary conditions of narcissistic leaders under different cultural backgrounds firstly. Then, this study promotes the research of the “team followership” topic, which has not received much attention from researchers. Finally, it provides a theoretical reference for enterprises to reduce the negative effects of narcissistic leaders and improve followership of employees.

## Literature Review and Hypotheses Development

### Narcissistic Leaders and Followership of Subordinates

Followership is the behavioral response of subordinates to superiors and their relationship. It arises from the interaction between leaders and followers, and is reflected in the attitudes, behaviors, and abilities of the followers (Kirkman et al., 2009; Derue et al., 2011). If subordinates perceive the dark side or improper supervision of the superior narcissistic leaders, there will be a series of negative influences, which may change their following behaviors. Liu and Wang (2013) found that abusive supervision and supervisor–subordinate guanxi (SSG) are negatively correlated (Liu and Wang, 2013). The narcissistic leaders only care about themselves, who show no sympathy to the subordinates, and often use fraudulent approaches to satisfy self-interest or adopt improper supervisory actions in order to protect own interests and authority (Nevicka et al., 2018). In those cases, it is difficult for subordinates to establish a supportive relationship with the leader. According to social distance theory of leadership, when the social distance between the leader and the followers is closer, they will judge and evaluate the leader based on their personal experience and perception to a higher degree (Schaubroeck et al., 2007; Nevicka et al., 2018). We mainly discuss the influence of narcissistic leaders on subordinates. Since subordinates have more opportunities to communicate and contact with their direct leaders, they can observe and perceive the narcissistic traits and behaviors of the leaders more thoroughly, and the negative or destructive elements that narcissistic leaders bring are also more likely to be exposed to subordinates (Nevicka et al., 2018). Thus, it can be inferred that the narcissistic leader has a certain destructive effect on the followership of subordinates. Therefore, we have proposed the following hypothesis:

*H1: Narcissistic leaders have negative impact on fellowership of subordinates.*

### Narcissistic Leaders and Team Followership

Team followership is defined as an in-role positive behavior implemented by a follower group that is conducive to improving the effectiveness of leadership, which is derived from the common perception and identification of team leaders in the team leadership process (Cao et al., 2019). Tee et al. (2013) argued that the characteristics of individual followers will be brought together at the team level through social identification, forming team characteristics or team behaviors, further affecting leadership characteristics or behaviors. The formation of team followership is affected by the joint action of team-level leaders and each follower in the team. It is the integration of a series of behaviors, including team progress, team recognition, team execution ability, and team relationship that team members conduct as a whole toward the team goal and in the interaction with leaders (Epitropaki et al., 2016; Cao et al., 2019). Therefore, the leader-oriented team followership is, actually, the reflection of the relationship between the leader and the followers. In team followership, leadership is an external factor that triggers following behavior. To some extent, team followership reflects how much the team supports and trusts the leader. Through this collective behavior, leadership effectiveness is improved (Fairfield, 2007). However, narcissistic leaders and their team have high visibility due to the needs of work, so their stubbornness, lack of cooperation, and other negative characteristics will be fully exposed to the team members (Nevicka et al., 2018), which is obviously not conducive to relationship commitment of the team to the leader. Therefore, it can be inferred that, to some extent, the narcissistic leaders have a negative impact on team followership.

*H2: Team narcissistic leaders and team followership are negatively correlated.*

### The Mediating Role of the “Leader-Subordinate” Relationship and the “Leader-Team” Relationship

#### The Mediating Role of Supervisor–Subordinate Guanxi

Supervisor–subordinate guanxi emphatically refers to the personal relationship between the leader and the subordinates, including the private communication and emotional interaction between the two parties outside of work (Cheung et al., 2009). Trait theory of leadership states that leadership traits will be reflected in the interpersonal interaction between the leader and the subordinates, affecting the quality of their interaction, and further affecting the behavior and attitude of the subordinates (Derue et al., 2011). The social communication and interaction between subordinates and leaders are the key to organizational management. Studies have found that the quality of SSG has a direct impact on job satisfaction, extra-role behaviors, job performance, and turnover intention of subordinates (Zhang et al., 2015). The social distance theory of leadership believes that, in a closer relationship, destructive leaders with a negative side have a greater negative impact on the work attitude of subordinates (Nevicka et al., 2018). In supervisor–subordinate guanxi, the narcissistic leaders have a capricious, erratic, and irritable personality, and the subordinates are often criticized and threatened. This leads to the rise of negative emotions in subordinates, making them unable to perceive the respect and care that they deserve. Therefore, it is difficult for the narcissistic leaders and the subordinates to reach emotionally close status (Judge et al., 2006). Followership can be seen as a return from the subordinates to the superiors. How followers show following behavior is closely related to their emotional orientation after the cognitive experience. Law et al. (2000) found that, when supervisor–subordinate guanxi is better, subordinates will have more trust and better personal feelings for the leader, and the leader will also show more care and attention to the work and the life of the subordinates. To some extent, the willingness and pleasure of cooperation between the two parties are increased (Law et al., 2000). On the contrary, when the supervisor–subordinate guanxi is negative, the willingness of both parties for cooperation will be decreased, and the willingness of the subordinates to follow will be decreased. Therefore, the narcissistic leaders can be destructive to supervisor–subordinate guanxi, and the followership of subordinates can be influenced through supervisor–subordinate guanxi. Thus, we have proposed Hypothesis 3.

*H3: Supervisor–subordinate guanxi mediates the negative relationship between narcissistic leaders and followership of subordinates.*

### The Intermediary Role of Team Relational Identification With Leaders

Team relational identification (RI) with leaders is a higher-level content constructed by RI. It is essentially a larger-scale role identification and relationship recognition. The more recognized the collective is, the stronger the role relationship between the two parties (Zhu et al., 2013). Hu and Judge (2017) believed that the relational identification of team leaders is an important relationship process, which directly measures the psychological intimacy between team members and leaders. Team relational identification reflects the extent to which team members define themselves based on their relationship with the leader (Sluss and Ashforth, 2007), and they often focus on role relationships and comparison with a standard role. Research has found that characteristics of leaders, such as extroversion, pleasantness, and sense of responsibility, have a positive impact on the relational identification with leaders at the team level (Hu and Judge, 2017). Narcissistic leaders are often considered to have a temperamental personality, who often show indifference, self-centeredness, and lack of teamwork (Hoffman et al., 2011). And that does not conform to the positive perception of team members for their relationship with the leaders. Although the narcissistic leaders are sometimes considered to be bold, confident, and dominated, the characteristics of the leaders, such as arrogance, self-interest, high possessiveness, and desire, for control will be fully perceived by team members due to the low social distance, which may influence the positive effects (Back et al., 2010). From the above discussion, it can be inferred that, when the team leader is a narcissistic leader, the negative characteristics of narcissism will hinder the formation of a good relationship between the team and the leader. Even the narcissistic leader is in a high position, it is difficult to form high-quality relational identification with the leader, and the leadership role will not be internalized by the team, which affects team followership to some extent. Therefore, the following hypothesis is proposed.

*H4: Team relational identification with the leader mediates the negative relationship between team narcissistic leaders and team followership.*

### The Moderating Role of Individual and Team Values

#### The Moderating Role of Traditionality

According to the leader trait process model, a leadership trait will have a good or bad impact on the organization or employee behavior and will be affected by the individual characteristics of employees (Hu and Judge, 2017). As a personality characteristic variable that can reflect differences in individual values, personal traditionality has an important impact on the attitudes and behaviors of employees (Chen and Aryee, 2007). The dominance complementarity theory believes that effective and continuous interpersonal relationships need to be complemented by domination and obedience (Hardert and Carson, 1969; Kiesler, 1983). In other words, if both parties accept that one party plays the dominant, controlling role, while the other plays a submissive, docile role, a satisfying and productive relationship will then be promoted (Chen et al., 2016). Compared with leaders with other characteristics, narcissistic leaders are dedicated to chasing power, they are eager to be worshipped, and tend to be tougher in their attitude toward subordinates. However, individuals with high traditionality have deep-rooted thoughts of superiority and inferiority, making it easy for them to regard leaders as an authority and develop dependence even worship (Farh et al., 1997).

According to the dominance complementarity theory, if a goal of one party in social interaction is to dominate the other party, it will facilitate a better interaction if the other party adopts a more submissive behavior (Kiesler, 1983). Through the above analysis, narcissistic leaders and high-traditionality subordinates can achieve the complementary effect of dominance complementarity to some extent, therefore reducing the uncertainty in the social interaction between the two parties and avoiding unnecessary competition and conflict. This will reduce the negative impact of narcissistic leaders on subordinates to some extent, especially reduce their destructive effect on supervisor–subordinate guanxi. Based on the above analysis, we have proposed the following hypothesis.

*H5: Traditionality moderates the relationship between narcissistic leaders and supervisor–subordinate guanxi. When the traditionality is high, the negative relationship between the narcissistic leaders and supervisor–subordinate guanxi is weak.*

#### The Moderating Effect of Team Power Distance

Team power distance refers to the basic attitudes of team members toward power, control, authority, and obedience, including the aggregation of individual values at the team level (Cole et al., 2013). Team values reflect the acceptance degree of most team members toward the differences of leaders or team position and power (Earley, 1999). Team members’ acceptance of power inequality will be an important factor affecting the effectiveness of narcissistic leadership (Daniels and Greguras, 2014). The team power distance determines whether the behavior of the leader is appropriate, whether it is accepted by the team, and whether the team will submit to the will of the leader (Yang et al., 2007). According to the dominance complementarity theory, a leader who is willing to dominate and a team that is willing to obey can complement each other and reduce the conflict, achieving a better dominance complementarity effect. One of the typical traits of narcissistic leaders is the extreme desire for power and self, and they show a strong desire to dominate employees (Grijalva and Harms, 2014). In addition, the self-confident and outgoing personality traits also, to some extent, meet the expectations for leaders of a team with high-power distance. We, therefore, conclude that narcissistic leaders are easier to be accepted in a team with a high team-power distance, where conflict and discomfort can be reduced. On the contrary, a team with low-power distances shows less desire to obey; they prefer leaders to share power and hope to participate in decision-making, expecting more control over tasks (Loi et al., 2012). That is obviously incompatible with the behavioral style of the narcissistic leader, leading to the increase of conflict between the leader and the team to some extent. In such a team, although the narcissistic leaders are in the leadership position, they are difficult to get recognition from the team, let alone the internalized leadership and relationship recognition. Research by Farh et al. (2007) also shows that traditional culture and power distance have an important regulatory effect on the evaluation of organizational support of employees, and leadership behavior can be seen as a form of organizational support. Therefore, we put forward:

*H6: Team power distance moderates the negative relationship between team narcissistic leaders and team relational identification with the leader. Such negative relationship is weakened when team power distance is high rather than low.*

**Figure 1:**
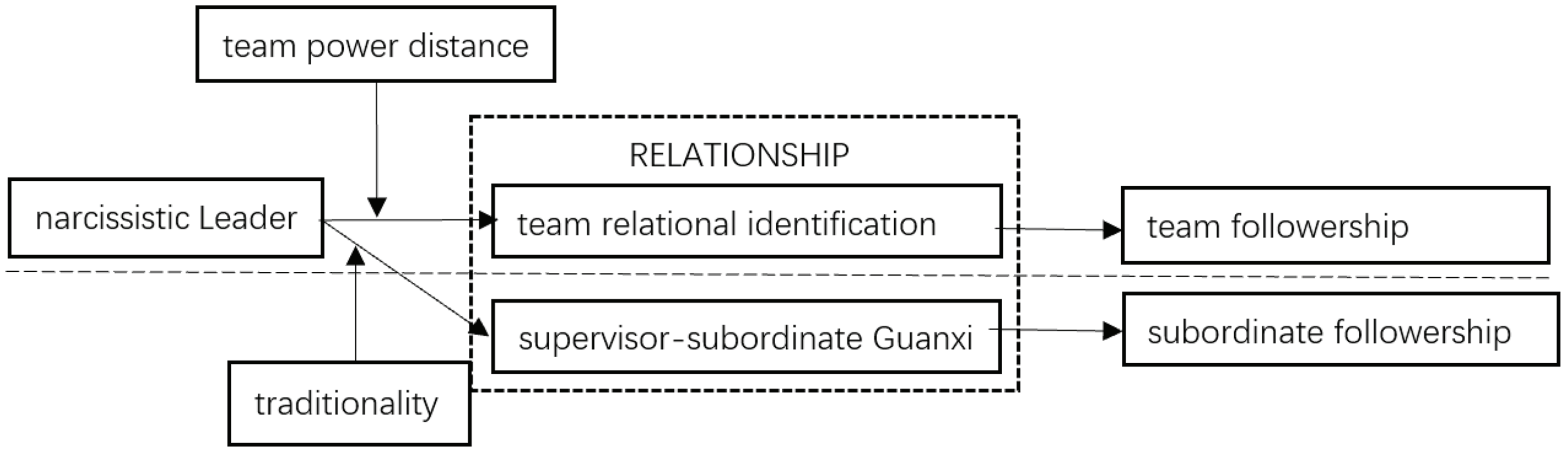
Research framework.

The theoretical model of the study is shown in Figure 1 below.

## Methodology

### Sampling and Procedure

Purposive sampling was adopted in the research. Our target participants include employees working in the big private companies, which are mainly involved in the manufacturing and technology industry in China. In terms of the survey personnel, four survey coordinators assisted in collecting data in their respective units. Using the employee ID lists provided by HR departments, the coordinators coded the questionnaires for matched supervisor and employee surveys.

We conducted a two-phase questionnaire survey in order to control common method deviation as much as possible and reflect the influence of independent variables on dependent variables. In phase 1, supervisors were asked to assess the narcissistic personality, and subordinates were asked to assess “supervisor–subordinate guanxi,” “team relational identification with leaders,” “team power distance,” and “traditionality.” We attached a cover letter to explain the purpose of our study and to emphasize the voluntary nature of the research. In order to ensure confidentiality, we asked each respondent to place his/her completed questionnaire into a sealed envelope to be collected by one of the researchers. We received 139 completed pieces of questionnaires for supervisors matched with 457 questionnaires for subordinates, representing the response rate of 87.0% in phase 1.

One month later (phase 2), we conducted the second-phase survey, following the same procedures as in phase 1. Supervisors who were tested a month ago finished the “team followership” questionnaire. The employees who previously filled out the questionnaire were asked to provide their ratings on “followership of themselves.” Each piece of the questionnaire was marked with a unique code, which was recorded in a master file such that the responses received from the two phases can be matched. Questionnaires with too many omitted items and less than three team members were removed, we finally got matching questionnaires for 103 leaders and 336 subordinates which overall response rate of 78.2%. Within the sample of the supervisors, 72.5% were males, the average age was 35, and the average working experience was 7 years. As for the employees, 61.3% were males, and 38.7% were females; the average age of employees was 28.1; and the average working experience was 3 years. Most of the members (86.7%) attained a college-level education or above. Team size, excluding team leaders, ranged from two to eight members with a mean of four members.

“Harman single factor test” was used to detect common method deviations. The results show that for the data of narcissism and team followership questionnaire filled by leaders, factor analysis has obtained a total of seven common factors with eigenvalues greater than 1, which explains 68.77% of the variation, and the proportion of the first principal component is 29.84%, indicating that the scale data filled by leaders have no serious homologous error. When the questionnaire data for the employees is not rotated, eight common factors with eigenvalues greater than 1 are obtained by factor analysis, which explains that 66.87% of the variation and the proportion of the first principal component is 29.67%. The largest factor cannot explain most of the variation, indicating that there is no serious homologous error in the employee data of this study.

### Measures

Each measure has a response scale from 1 = strongly disagree to 5 = strongly agree, except for narcissistic leaders. All measures used in this survey are adopted from the established scales. Considering that all of our participants are Chinese, we went through appropriate back translation procedures (Brislin, 1970) to develop the Chinese version for the measures.

#### Narcissistic Leader

We assessed narcissistic leaders by using the NPI-16 scale (Ames et al., 2006). This questionnaire is self-evaluated by the leader. Sample items from this scale include the following (the first item in each pairing reflects narcissism): “I am an extraordinary person” or “I am much like everybody else.”

#### Followership

The 21-item followership scale is adopted, developed by Zhou et al. (2015) based on the Chinese context. Sample items are “I admire and learn from the ability of the leader in business and management, etc.”

#### Team Followership

We used the 21-item followership scales from Zhou et al. (2015) to measure team followership. Moreover, the wording is modified from “my supervisor” to “my team members” to reflect a relevant referent shift.

#### Supervisor–Subordinate Guanxi

We used the six-item scales from Law et al. (2000) to measure supervisor–subordinate guanxi. The scale adopts Chinese employees as a sample and involves emotional interactions between superiors and subordinates.

#### Team Relational Identification With Leaders

The measurement of team relational identification with leaders selects the 10-item scale developed by Walumbwa and Hartnell (2011). Moreover, wording is modified from “my supervisor” to “my team leader” to reflect a relevant referent shift.

#### Team Power Distance

The eight-item scale developed by Kirkman et al. (2009) is adopted. Drawing on the previous study, ratings of individual members of a team power distance value are aggregated to form a team-level power distance value (Hu and Judge, 2017).

#### Traditionality

We used the five-item scales from Yang (2003) to measure the traditionality of subordinates.

#### Control Variables

According to previous studies, we controlled for the effects of the gender, tenure of subordinates, and team size in the data processing (Eagly and Karau, 2002; Foster et al., 2003). This study uses the above variables as control variables.

## Results

### Analytical Approach

Employees in our sample were grouped within their branches, each headed by a manager. In order to appropriately model this nested nature, we used multilevel data modeling (Raudenbush and Bryk, 2002). We used hierarchical linear modeling (HLM) version with a restricted maximum likelihood estimation method for the analysis. In addition, HLM is effective for modeling cross-level interaction effects between group-level predictors and individual-level independent variables on outcome variables (Hofmann et al., 2003). Following convention, we used group-mean centering (Hofmann and Gavin, 1998; Enders and Tofighi, 2007).

In our study, the variables “team power distance” and “team relational identification with leaders” refer to aggregates of individual responses to the team level of analysis. Aggregation is justified by theoretical as well as empirical arguments (Rousseau, 1985) and was a critical requirement to demonstrate high within-team agreement in order to justify using the team average as an indicator of a team-level variable (Fredrickson, 1984). In order to empirically justify aggregating individual scores to the group, we calculated within-group agreement, intra-class correlations (ICC1), and the reliability of the means (ICC2; Klein and Kozlowski, 2000). The mean Rwg value of team power distance and team relational identification with leaders are 0.873 and 0.913, well above the conventional cut-off value of 0.70 (Fredrickson, 1984). And we obtained acceptable ICC values [ICC1_(PD)_ = 0.337, ICC1_(RI)_ = 0.451, ICC2_(PD)_ = 0.742, and ICC2_(RI)_ = 0.823]. Thus, the results have provided support for aggregating the individual responses to the team level to represent power distance and relational identification of each team with leaders (Table 1).

**Table 1:** Data aggregation test results.

	RWG	F	ICC1	ICC2
PD	0.873	3.879^***^	0.337	0.742
RI	0.913	5.652^***^	0.451	0.823

*** *p* < 0.001.

*n* = 336; F, followership; RI, team relational identification with leaders; and PD, team power distance.

The Cronbach α is used to measure the internal consistency reliability of the questionnaire as a whole and each item. The Cronbach α of each scale is greater than 0.7 [α_(F)_ = 0.921, α_(SSG)_ = 0.883, α_(RI)_ = 0.939, α_(T)_ = 0.865, and α_(PD)_ = 0.899]. Respectively, it can be seen that the reliability coefficient of each scale is greater than 0.7, indicating that each dimension has good internal consistency.

### Descriptive Statistics

Descriptive statistics, reliabilities, and correlations are provided in Table 2. Reliabilities reported on the diagonal show strong internal consistency across all measures. Narcissistic leaders and team followership are significantly negatively correlated (*r* = −0.41, *p* < 0.01), and a narcissistic leader has significant negative correlation with team relational identification (*r* = −0.27, *p* < 0.01). SSG has a positive relationship with followership (*r* = −0.40, *p* < 0.01). Team relational identification has a significant positive impact on team followership (*r* = 0.47, *p* < 0.01).

**Table 2:** Means, SDs, and correlations of variables.

		1	2	3	4	5	6
L1	L-gender	1					
	T-size	−0.086	1				
	N	0.177	0.048	1			
	PD	0.335^**^	0.004	−0.273^**^	1		
	RI	0.328^*^	0.101	−0.460^**^	0.523^**^	1	
	TF	0.017	0.089	−0.413^**^	0.510^**^	0.478^**^	1
	M	1.351	10.348	7.787	3.458	3.595	3.018
	SD	0.467	9.880	4.116	0.612	0.664	0.536
L2	E-gender	1					
	E-tenure	−0.050	1				
	T	0.028	−0.108^*^	1			
	SSG	0.024	0.068	0.263^**^	1		
	F	0.015	0.086	0.268^**^	0.401^**^	1	
	M	1.391	1.853	3.607	3.683	3.721	
	SD	0.489	1.470	0.670	0.808	0.534^**^	

* *p* < 0.5;

** *p* < 0.01

*n* = 336; N, narcissistic leaders; F, followership; TF, team followership; SSG, supervisor–subordinate guanxi; RI, team relational identification with leaders; T, traditionality; PD, team power distance; L, leader; E, employee; T, team; L1, team level; and L2, individual level.

### Confirmatory Factor Analyses

Following the recommendation of Anderson and Gerbing (1988), we examined the construct validity of the variables before testing the hypotheses. We conducted a series of confirmatory factor analyses (CFA), using AMOS 20.0 to examine the distinctiveness of our study variables based on chi-square statistics and fit indices of RMSEA, CFI, and TLI. As shown in Table 3, the fit indices support that the hypothesized three-factor model at the team level has a good fit (χ^2^/df = 1.124, GFI = 0.898, NFI = 0.897, CFI = 0.988, and RMSEA = 0.020 < 0.080), indicating that this scale has good structural validity. The main fitting indicators of the three-factor model at the individual level are better than the single-factor model (χ^2^/df = 1.120, GFI = 0.915, NFI = 0.905, CFI = 0.989, and RMSEA = 0.019 < 0.08), suggesting that our respondents can distinguish the focal constructs clearly (Table 3).

**Table 3:** Confirmatory factor analysis (CFA) results.

	Model	Χ^2^	df	Χ^2^/df	SRMR	GFI	NFI	CFI	RMSEA
L1	One-factor (TF/PD/RI)	3707.656	629.000	5.895	0.132	0.456	0.451	0.494	0.123
	Two-factors (PD/RI+TF)	1738.521	622.000	2.795	0.092	0.676	0.745	0.818	0.074
	Three-factors (PD+RI+TF)	696.686	620.000	1.124	0.042	0.898	0.897	0.988	0.020
L2	One-factor (S+F)	2682.457	464.000	5.781	0.116	0.577	0.500	0.544	0.120
	Two-factors (SSG/T+F)	1149.048	457.000	2.514	0.081	0.779	0.786	0.858	0.068
	Three-factors (F+T+SSG)	509.717	455.000	1.120	0.042	0.915	0.905	0.989	0.019

*n* = 336; N, narcissistic leaders; F, followership; TF, team followership; SSG, supervisor–subordinate guanxi; RI, team relational identification with leaders; L, leaders; E, employee; T, team; L1, team level; and L2, individual level.

#### Hypothesis Testing

We used HLM (Bryk et al., 1996) for statistical analysis to solve the problem of sample independence. A zero-model test was performed on SSG and followership variables. We obtained acceptable values [U_(SSG)_ = 0.304, R_(SSG)_ = 0.361, ICC_(SSG)_ = 0.457, χ^2^_(df)_ = 357.804, and *p* < 0.001]. The intergroup variation accounts for 45.7% of the total variation, indicating significant differences between the groups. Similarly, the intergroup variation of followership accounts for 55.4% of the total variation, indicating that subsequent cross-layer analysis can be performed [U_(F)_ = 0.160, R_(F)_ = 0.129, ICC_(F)_ = 0.554, χ^2^_(F)_ = 490.584, and *p* < 0.001; Table 4].

**Table 4:** Zero model test.

	SSG	F
	M0	
	3.671^***^	3.709^***^
R(Sigma_squared)	0.361	0.129
U(Tau)	0.304	0.160
ICC	0.457	0.554
Chi-square	357.804^***^	490.584^***^
Deviance	734.032	419.543

*** *p* < 0.001.

*n* = 336; F, followership; and SSG, supervisor–subordinate guanxi.

### Narcissistic Leaders and Followership

After controlling relevant variables such as demographics, our study performed multiple linear regression analysis on the data. It can be seen from Table 5 that narcissistic leaders have a significant negative impact on followership and team followership (*β* = −0.074, *p* < 0.001; *β* = −0.75, *p* < 0.01), thus, our Hypothesis 1 and Hypothesis 2 have been supported. Model 1 mainly includes independent variables (narcissistic leaders) and dependent variables.

**Table 5:** Results of mediating regression tests.

	F		SSG	TF		RI
	M1	M2	M3	M1	M2	M3
Intercept	3.708^***^	3.709^**^	3.668^***^	3.711^***^	3.511^**^	3.710^***^
Control variables						
E_gender	−0.011	−0.017	0.073	−0.008	−0.020	−0.003
E-tenure	0.012	0.025	0.025	0.019	0.018	0.010
L-gender	0.090	0.062	0.246	0.087	−0.038	0.262^*^
T-size_	0.001	0.005	−0.003	0.004	−0.003	0.154
Independent variable						
N	−0.074^***^	−0.068^***^	−0.057^**^	−0.075^**^	−0.053^**^	−0.413^**^
Mediators						
SSG		0.115^***^				
RI					0.331^**^	
R(Sigma_square)	0.132	0.137	0.362	0.133	0.134	0.305
U(Tau)	0.060	0.038	0.248	0.059	0.022	0.245
ICC	0.313	0.218	0.407	0.307	0.141	0.155
Chi-square	215.191^***^	162.875^***^	286.676^***^	210.140^***^	127.626^***^	428.101^***^
Deviance	425.938	418.341	770.710	417.984	385.417	489.190

* *p* < 0.05;

** *p* < 0.01;

*** *p* < 0.001.

*n* = 336; N, narcissistic leaders; F, followership; TF, team followership; SSG, supervisor–subordinate guanxi; RI, team relational identification with leaders; L, leader; E, employees; and T, team.

### The Mediating Regression Tests

The mediating regression tests are reported in Table 5. Model 1(F) and Model 1(TF) show that a narcissistic leader has a negative relationship with followership (*β* = −0.074, *p* < 0.001) and team followership (*β* = −0.75, *p* < 0.01). Model 3 shows the negative relationship between narcissistic leaders and SSG (*β* = −0.057, *p* < 0.001), and negative relationship between a narcissistic leader and team RI (*β* = −0.413, *p* < 0.01). Thus, the first two conditions of our mediation hypothesis are also met. To examine the third condition of the mediation, we regressed SSG on followership and RI on team followership with the effect of narcissistic leaders controlled (Model 2). The results show the positive relationship between SSG and followership (*β* = 0.115, *p* < 0.001), and the positive relationship between RI and team followership (*β* = 0.331, *p* < 0.01), and the negative effect of narcissistic leaders on followership and team followership reduced (*β* = −0.068, *p* < 0.001; *β* = −0.053, *p* < 0.01), thus indicating a partial mediation effect. Hypothesis 3 and Hypothesis 4 are supported.

### The Moderating Regression Tests

**Figure 2:**
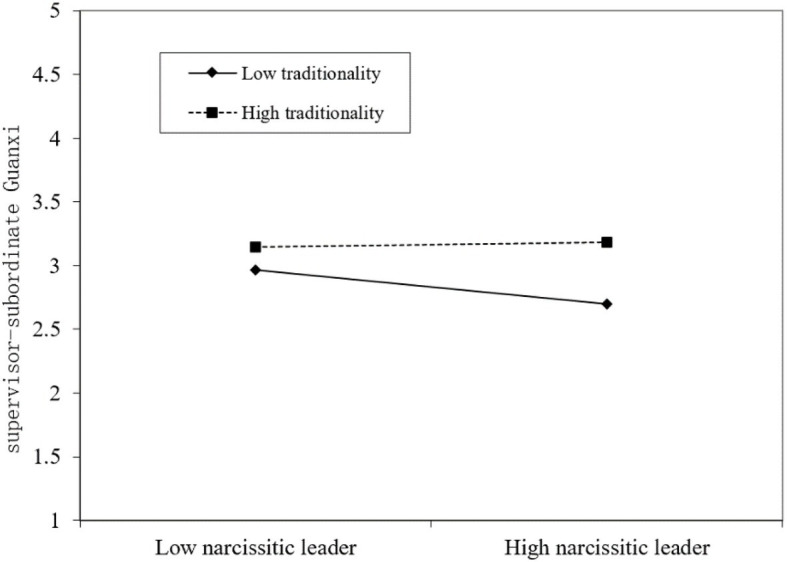
Moderating effect of traditionality.

**Figure 3:**
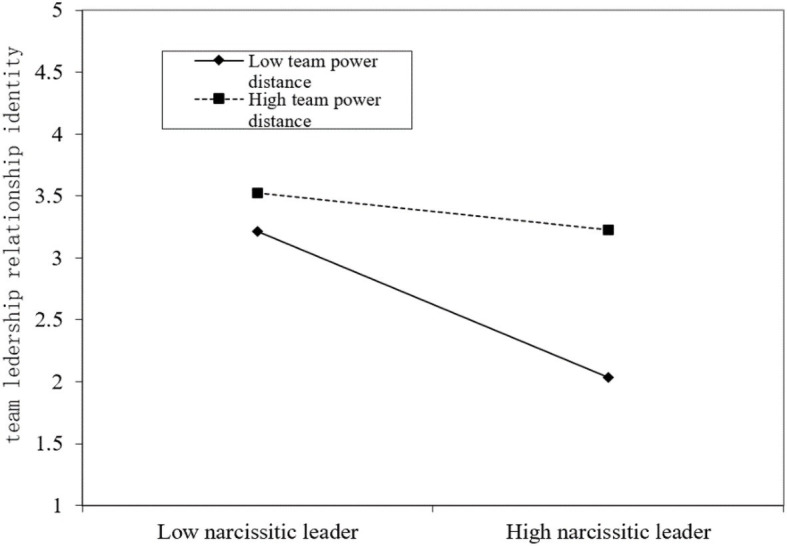
Moderating effect of team power distance (PD).

Table 6 shows the results of examining whether traditionality plays a moderate role between the negative relationship between narcissistic leaders and SSG, and whether team PD moderates the negative relationship between narcissistic leaders and team RI. The interaction term is significant (*β* = 0.076, *p* < 0.001; *β* = 0.221, *p* < 0.01), and the percentage of variation between groups is reduced, further proving that SSG and RI have significant regulatory effects. In order to further prove that the moderating effects are as expected, we draw a diagram of the moderating effect as shown in Figures 2, 3.

**Table 6:** Results of moderating regression tests.

	SSG			RI		
	M1	M2	M3	M1	M2	M3
Intercept	3.668^***^	3.669^***^	3.710^***^	3.712^***^	3.713^***^	3.513^**^
Control variables						
E_gender	0.073	0.077	0.058	−0.003	−0.007	−0.019
E-tenure	0.025	0.025	0.035	0.010	0.019	0.018
L-gender	0.246	0.216	0.139	0.262^*^	0.153	0.111
T-size_	−0.003	−0.003	−0.004	0.154	0.085	0.054
Independent variable						
N	−0.057^**^	−0.054^**^	−0.063^***^	−0.413^**^	-	-
Moderator						
T		0.109	0.166^*^			
PD					0.376^**^	0.407^**^
Interaction						
N×T			0.076^***^			
N×PD						0.221^**^
R(Sigma_square)	0.362	0.368	0.380	0.305	0.429	0.472
U(Tau)	0.248	0.224	0.133	0.245	0.371	0.412
ICC	0.407	0.378	0.259	0.155	0.123	0.043
Chi-square	286.676^***^	262.418^***^	183.447^****^	428.101^***^	420.58^**^	220.49^**^
Deviance	770.710	770.126	759.315	489.190	466.98	415.70

* *p* < 0.05;

** *p* < 0.01;

*** *p* < 0.001.

*n* = 336; N, narcissistic leaders; SSG, supervisor–subordinate guanxi; RI, team relational identification with leaders; T, traditionality; PD, team power distance; L, leader; E, employee; and T, team.

Consistent with our expectation, the negative relationship between narcissistic leaders and SSG is relatively weakened for a subordinate who has high traditionality (T), and the negative relationship between narcissistic leaders and RI is relatively reduced when team PD is higher. Thus, our Hypothesis 5 and Hypothesis 6 are supported.

## Conclusion and Discussion

Although scholars have illustrated in theoretical research that narcissistic leaders will have a significant impact on the work achievement of subordinates, the research on the mechanism of narcissistic leaders is not in-depth. The team-level impact of narcissistic leaders is less involved. There are a few studies focusing on narcissistic CEOs and team performance (Benson and Hogan, 2008; Russell and Stone, 2013), while the discussion about the influence of team narcissistic leaders on team attitude and behavior is lacking. Scholars have mostly explored the mechanism of narcissistic leaders from the perspective of leadership behavior (Braun, 2017) and paid little attention to the leader-subordinate interpersonal relationship and the leader-team relationship perspectives.

We analyzed the effect pathway that narcissistic leaders influence subordinates and team followership by affecting supervisor–subordinate guanxi and team relational identification with leaders. We verified the regulatory role of individual and team values based on the dominance complementarity theory, which reflects the importance of complementary traits between leaders and employees as well as between leaders and teams (Post, 1986). Our study can also work as a supplement to previous research. The research results show that narcissistic leaders have a certain negative influence on both subordinates and team followership. Supervisor–subordinate guanxi and team relational identification with leaders play a part of an intermediary role at the individual level and the team level, respectively. Individual and team values play a regulatory role in this process. Subordinate traditionality regulates the negative impact of narcissistic leaders on supervisor–subordinate guanxi. Team power distance plays a regulatory role between the identification of narcissistic leaders and team leaders. The higher the team power distance, the lower the negative impact narcissistic leaders have on team relational identification with leaders.

The research results also have verified that the negative effects of narcissistic leaders considered by previous scholars are far greater than their positive effects. However, due to the complex nature of narcissism, it is difficult for us to prevent narcissists from becoming leaders (Nevicka et al., 2013), which requires us to study narcissists from a more detailed perspective (Owens et al., 2015). Also, we remind the organization managers to be alert to the harm of narcissistic leaders and reduce their negative impact through appropriate subordinates and a team match. Especially under the background of Chinese culture, the culture of “harmony” has always been emphasized. On the one hand, it pays attention to the harmony of relations; on the other hand, it advocates authority and emphasizes the priority in rank. Our research results reflect such a cultural feature to some extent. A narcissistic leader, as a kind of a leader with dark traits, has a certain negative influence on employee behavior. However, for the subordinates with high traditionality, this negative effect was significantly weakened. Affected by the sense of hierarchy in Chinese traditional culture, subordinates with high traditionality are more willing to obey authority and leaders, and they also show higher acceptance of some unreasonable leadership behaviors. For individuals with high traditionality, narcissists who are confident and assertive may be considered a positive leader full of power (Watts et al., 2013).

### Theoretical Implications

We discussed the intermediary mechanism of the influence of narcissistic leaders on individuals and teams from the perspective of “guanxi.” Based on the Chinese cultural background, we expanded the related research about the influence of narcissistic leaders on the attitude and behavior of subordinates. Chinese society has always emphasized “guanxi.” In the interactions of subordinates with leaders, in addition to the principle of fairness and reciprocity, they pay more attention to the principles of “guanxi” (Chen and Chen, 2004). Therefore, the supervisor–subordinate guanxi is more sensitive than the western countries, and it has a more obvious influence on the attitudes and behaviors of organization members (Farh et al., 1998). Most of the existing studies are based on the contractual relationship of equal exchange (Liao et al., 2019; Bernerth, 2020), which explores the mechanism of the influence of narcissistic leaders on individual employees and ignores the “personal relationship” and “relational identification with leaders.” The study on the influence of narcissistic leaders at the team level is also insufficient. Based on the “guanxi” perspective of the Chinese cultural background, our research explores the influence of supervisor–subordinate guanxi that narcissistic leaders establish through informal social interactions and the influence of team relational identification with leaders on subordinates followership and team followership, and further broadens the scope and the theoretical level of narcissistic leaders research.

Based on the dominance complementarity theory, we discussed the boundary conditions of the influence of the narcissistic leaders. Narcissistic leaders are known as leaders with complex personality traits. Research of scholars on their impact is not completely consistent. Although most studies have proved the negative impact of narcissistic leaders on employees, teams, and organizations, as well as some boundary conditions of the negative impact (Huang et al., 2019), which team factors can reduce the negative effects have received little attention and study. To some extent, our study reflects the importance of traits complementarity between leaders and teams, especially the regulatory role of individual traditionality and team power distance, which reflect Chinese cultural values.

We promote research on the emerging topic “team followership.” Followership is a hot topic in current business management, but followership research mainly focuses on the individual followership in an organization, while it ignores the exploration of team followership (Leroy et al., 2015; Cao et al., 2019). With the increase of uncertainty in the external environment, it is difficult for companies to create brilliant performance by working alone, and the role of a team in the organization is becoming more and more important. To some extent, team followership reflects the degree of team support and trust that the leader obtains, and the relationship between leaders and teams also directly affects team performance (Hu and Judge, 2017). Compared with the individual following, team followership at work has higher research and practical value. Therefore, scholars call for more research on followership at the team level (Uhl-Bien et al., 2014). But, at present, team followership is still a relatively new topic; related empirical research is rare. Therefore, our research has promoted related empirical research on team followership, to some extent, by exploring the influence of narcissistic leaders on team followership. Our research on team followership has expanded the level of research on followership and responds to the call of scholars to deepen the research on team followership. Our research has a certain significance for understanding the team leadership process and guiding the team management practice.

### Practical Implications

The rapidly changing world and economic situation make the company staff full of anxiety and challenges to both leaders and employees of a company. From a strategic point of view, how leaders facilitate their own advantages enhance confidence and sense of security of employees, enable employees and leaders to work together, and help each other is extremely important for reducing internal turbulence and enhancing organizational resilience. Followership, as a product of the interactions between leaders and subordinates, directly influences the effectiveness of leadership performance and the achievement of strategic goals of an organization. This paper has the following inspirations for company management: firstly, strengthen the understanding of narcissistic leaders during the crisis period and provide corresponding measures for enterprises to avoid the negative effects of narcissistic leaders and take advantage of narcissistic leaders. Through the discussion of the boundary conditions in the process of narcissistic leader influence, we provide a reference for the organization to select leadership and team management in times of crisis. In addition, we provide a reference for companies to improve employees and team followership in order to strengthen team construction and management. Companies should pay full attention to the influence of own characteristics of leaders on employees and teams. In team construction, they should fully consider the matching of leadership and team values, as well as the changes in the external environment so that leaders and teams can complement the strengths of each other.

### Research Limitations

We conducted a questionnaire survey in a two-time period, but it cannot guarantee a good evaluation of the causal relationship between variables. Future research can make a more rigorous test on the research problems through longitudinal data with a large time span or through the introduction of experimental research and other methods so as to make the relationship between related variables more convincing.

The measurement of team followership is based on the scale of individual followership. While the connotation of team followership is not limited to the aggregation of individual followership. In the future, we still need to further develop the accurate scale to better reveal the concept of team followership and further promote the empirical research on team followership.

For narcissistic leaders, they may have different effects under some special circumstances or in the face of different subordinates and teams with different characteristics. Future research can explore the combined effect of these factors so as to verify the positive role of narcissistic leaders. It is well-known that COVID-19 strongly impacts mental response of workers and their well-being. It could be meaningful to consider the impact of some crisis events like COVID in the future study. Also, productivity has been considered as an outcome of perceived social support; higher perceived workplace support is independently associated with higher work productivity (Chen et al., 2016). Social support may ameliorate the negative effects of the crisis; organizations that provide technical support and emotional support for employees in a crisis can benefit by promoting better attitudes and behaviors (Vaziri et al., 2020). Future research can focus on the consequences of trust and support from subordinates and teams to leaders, and support from leaders to subordinates and teams.

## Data Availability Statement

The raw data supporting the conclusions of this article will be made available by the author, without undue reservation.

## Ethics Statement

The studies involving human participants were reviewed and approved by University of Taipei. The patients/participants provided their written informed consent to participate in this study.

## Author Contributions

The author confirms being the sole contributor of this work and has approved it for publication.

### Conflict of Interest

The author declares that the research was conducted in the absence of any commercial or financial relationships that could be construed as a potential conflict of interest.
